# Novel SNP Discovery in African Buffalo, *Syncerus caffer*, Using High-Throughput Sequencing

**DOI:** 10.1371/journal.pone.0048792

**Published:** 2012-11-07

**Authors:** Nikki le Roex, Harry Noyes, Andrew Brass, Daniel G. Bradley, Steven J. Kemp, Suzanne Kay, Paul D. van Helden, Eileen G. Hoal

**Affiliations:** 1 DST/NRF Centre of Excellence for Biomedical Tuberculosis Research/MRC Centre for Molecular and Cellular Biology, Division of Molecular Biology and Human Genetics, Faculty of Health Sciences, Stellenbosch University, Cape Town, South Africa; 2 Institute of Integrative Biology, University of Liverpool, Liverpool, United Kingdom; 3 School of Computer Science, University of Manchester, Manchester, United Kingdom; 4 Molecular Population Genetics, Smurfit Institute of Genetics, Trinity College Dublin, Dublin, Ireland; 5 International Livestock Research Institute, Nairobi, Kenya; Auburn University, United States of America

## Abstract

The African buffalo, *Syncerus caffer*, is one of the most abundant and ecologically important species of megafauna in the savannah ecosystem. It is an important prey species, as well as a host for a vast array of nematodes, pathogens and infectious diseases, such as bovine tuberculosis and corridor disease. Large-scale SNP discovery in this species would greatly facilitate further research into the area of host genetics and disease susceptibility, as well as provide a wealth of sequence information for other conservation and genomics studies. We sequenced pools of Cape buffalo DNA from a total of 9 animals, on an ABI SOLiD4 sequencer. The resulting short reads were mapped to the UMD3.1 *Bos taurus* genome assembly using both BWA and Bowtie software packages. A mean depth of 2.7× coverage over the mapped regions was obtained. Btau4 gene annotation was added to all SNPs identified within gene regions. Bowtie and BWA identified a maximum of 2,222,665 and 276,847 SNPs within the buffalo respectively, depending on analysis method. A panel of 173 SNPs was validated by fluorescent genotyping in 87 individuals. 27 SNPs failed to amplify, and of the remaining 146 SNPs, 43–54% of the Bowtie SNPs and 57–58% of the BWA SNPs were confirmed as polymorphic. dN/dS ratios found no evidence of positive selection, and although there were genes that appeared to be under negative selection, these were more likely to be slowly evolving house-keeping genes.

## Introduction

Investigating genetic variation between individuals, populations or species provides the basis for understanding the heritability of traits and phenotypes, and provides researchers with the opportunity to study complex issues in conservation, disease susceptibility, molecular ecology and many other disciplines [1]. Animal models have long provided the foundation for the genetic analysis of complex traits, using gene knockouts, recombinant and transgenic animals. The advent of whole-genome sequencing and the subsequent assembly of the genomes of the domestic cow, mouse, rat and others provides a resource pool of genetic markers with which to work in subsequent studies [2], [3]. However, when studying a species for which limited sequence information is available, it is necessary to generate sequence data in order to identify the genetic variants present in the population. The discovery of genetic variants, in particular large-scale SNP discovery, identifies genetic markers that may have the power to answer multiple research questions, and can be performed using a wide variety of technologies [4], [5].

The development of next-generation sequencing technologies, such as the ABI SOLiD, Illumina GA and HiSeq and Roche 454 platforms, has enabled a faster and more cost-effective approach to generating sequence data and SNP discovery. Each of these platforms has its own particular chemistry and combination of template preparation, sequencing and data analysis, and thus each has its own advantages and disadvantages [6], [7]. Benefits of working with SNP data include the fact that they are abundant, distributed throughout the genome, are easy to score and can be used in high-throughput screening [1], [8]. The sequence data obtained using next-generation technologies are typically short sequence reads of approximately 50–750 bp. Whole genome shotgun or re-sequencing using short reads requires the alignment of millions of sequence reads to a high quality reference genome sequence. Once the reads have been mapped to the reference genome, nucleotide variation between the sample and the reference can be identified [4]. For most species that do not have a fully sequenced genome, this can be problematic. However, if the genome of a closely related species is available, this can be used as a reference, although the proportion of data that can be mapped to the genome may be significantly reduced, and the data obtained will indicate variation seen between species as well as between individuals of the same species [9]–[11]. Other considerations such as depth of coverage and quality must be taken into account, particularly when using this approach.

The African buffalo, *Syncerus caffer*, is one of the most abundant and ecologically important species of megafauna in the savannah ecosystem. There are three recognised subspecies of African buffalo – the Cape buffalo (*Syncerus caffer caffer*), the West African buffalo (*Syncerus caffer brachyceros*) and the Forest buffalo (*Syncerus caffer nanus*). A fourth subspecies, the Central African Savannah buffalo (*Syncerus caffer aequinoctialis*), has also been proposed. All individuals used in this study were *Syncerus caffer caffer*. African buffalo are an important prey species, as well as a host for a vast array of nematodes, pathogens and infectious diseases [12]. Some of the infectious diseases for which the African buffalo is a wildlife host are bovine tuberculosis (BTB), corridor disease and foot-and-mouth disease [13]. Their current distribution extends throughout much of sub-Saharan Africa, although within this range the distribution is fragmented and largely confined to protected areas. The most recent IUCN census data estimates the global African buffalo population to be approximately 900,000 [14]. Previous genetic studies on buffalo have focused primarily on population differentiation and genetic diversity, using markers such as microsatellites, mitochondrial DNA and Y chromosomal loci [15]–[17]. Large-scale SNP discovery in this species would greatly facilitate further research into the area of host genetics and disease susceptibility, as well as provide a wealth of sequence information for other conservation and genomics studies.

Our goals in this study were to perform next-generation sequencing and mapping of the African buffalo genome using two different software packages, implement large-scale identification of novel SNPs within the African buffalo genome, and determine and compare the SNP validation rates using fluorescent genotyping. This data can then inform future studies.

## Materials and Methods

### Ethics

The Stellenbosch University Animal Care and Use Committee (SU ACU) deemed it unnecessary to obtain ethical clearance for this study as the blood samples used for DNA extraction were collected under the directive of SANParks and KwaZulu Natal Wildlife for other purposes, and their use in the present study is incidental.

### Samples

EDTA blood samples from nine Cape buffalo from Hluhluwe iMfolozi Park, in the KwaZulu Natal province of South Africa, were obtained during an annual BTB test and cull operation. Genomic DNA was extracted from the blood samples using the Qiagen DNeasy Blood and Tissue Kit, according to the manufacturer's instructions. Equimolar amounts of DNA from four and five animals were made into two barcoded paired end libraries. Fragment libraries were constructed according to manufacturer's instructions using the SOLiD™ Fragment Library Construction Kit, and sequenced on a single plate of an ABI SOLiD4 sequencer (Lifetech) to generate 50 bp reads.

### Mapping and SNP detection

Due to the absence of an assembled African buffalo reference genome, the *Bos taurus* UMD3.1 genome sequence, produced by the Centre for Bioinformatics and Computational Biology (CBCB) at the University of Maryland, was used as the reference genome in this study [18]. The short reads were mapped to the *Bos taurus* genome in colour space using two separate packages, BWA [19] and Bowtie [20]. SNPs were called from Bowtie reads with pileup and mpileup, and SNPs were called from BWA reads with pileup and Genome Analysis Tool Kit 1.1–3 (GATK) [21]. Reads were initially mapped with Bowtie with the following parameters -C (colorspace); –snpfrac 0.01 (1% of positions expected to be polymorphic); −3 1 (trim one base from the 3′ end of the read). SNPs were extracted from the reads mapped with Bowtie using pileup in SAMtools with the –c and –r 0.01 (1% of positions expected to be polymorphic) options [22]. 173 SNPs identified by this pipeline were submitted for validation. Subsequently the data was reanalysed using Bowtie with –snpfrac 0.001, followed by Picard Tools v1.48 to remove duplicates, and SAMtools mpileup with options -B (Disable probabilistic realignment); -d 29 (maximum depth 29) to identify SNPs. BWA was run with option -q 20 (trim low quality reads from 3′ end). SNPs were extracted from reads mapped with BWA with SAMtools using the same options as for Bowtie, and also with Picard Tools and GATK [21] for base quality score recalibration, indel realignment, duplicate removal, and SNP and INDEL discovery and genotyping [23]. Shellscripts for calling SNPs with GATK are available from the authors on request. Nucleotide differences called by SAMtools were classified as SNPs if (i) the alternate allele was supported by a minimum coverage of 2, and (ii) the alternate allele had a minimum Phred quality score of 20. Nucleotide differences called by GATK were classified as SNPs if they were flagged as “PASS”. The use of the *Bos taurus* reference genome in this study enabled the identification of two classes of SNPs - those that occur between the cow and buffalo, and those that occur within the buffalo population.

### Annotation

Gene annotation was added to the SNPs identified within gene regions, which were defined as from gene start to gene end, including introns. A local Perl script was then used to submit the SNPs within gene regions to the Ensembl v66 Application Programme Interface (API) to determine functional consequence [24], [25]. SNPs and annotations were written to a local MySQL database for storage and interrogation.

### Signatures of selection

The ratio of non-synonymous to synonymous substitutions (dN/dS) was calculated for all buffalo genes using PAML [26]. The Ensembl v66 API was used to identify the positions of SNPs within the coding sequence of bovine genes (*Bos taurus* genome build UMD3.1), and Cape buffalo SNPs were substituted into the gene coding sequences at these positions before submission to a local copy of PAML to obtain the dN/dS ratios. P values were obtained by taking twice the absolute difference between the log likelihood of the observed dN/dS ratio and the ratio obtained with dN/dS set to 1 and comparing it to χ^2^ with one degree of freedom. The Database for Annotation, Visualization, and Integrated Discovery (DAVID) was used to identify whether there were pathways that were overrepresented in the gene lists [27].

### Validation

A selection of 173 SNPs was made from the collective BWA pileup and Bowtie pileup SNP pool based on the predicted consequence and/or gene location of the SNP, in order to use the validation data in a subsequent case-control association study. SNPs were fluorescently genotyped in 87 Cape buffalo by KBioscience (www.kbioscience.co.uk), using their competitive allele-specific KASP SNP genotyping platform. Assays were designed for each of the SNPs by KBioscience. The KASP assay system is a competitive allele-specific PCR incorporating a FRET quencher cassette (www.kbioscience.co.uk). All but six of the SNPs validated as polymorphic within Cape buffalo were also validated in the original 9 individuals from the sequencing pool; the six SNPs not genotyped in the original samples were omitted due to technical reasons. KBioscience assay IDs are shown in Table S1 and are available to other users on application to KBiosciences.

## Results

### Mapping

The number of reads generated by the SOLiD sequencing was 442,984,249 in total. Sequence reads have been submitted to the sequence read archive at NCBI (accession number SRA051668.1). Given the relatively large genetic distance between *Bos taurus* and Cape buffalo, we initially used a high value of snpfrac (0.01) in the Bowtie parameters; this is the proportion of positions that are expected to be polymorphic. With snpfrac at 0.01, 23% of the reads mapped to the reference genome but the validation rate of the SNPs was relatively low (43%; see below). We therefore repeated the mapping with snfrac = 0.001, which reduced the percentage mapping to 21% and increased the validation rate to 54%. The mapped Bowtie reads were all 47 bases in length and covered 1.58GB, which is equivalent to 56% of the bovine genome being covered by at least one read, and represents 2.7× coverage of aligned data. Even fewer reads mapped with BWA (19%), of which 9.7% were PCR duplicates. The BWA reads were trimmed based on the base quality; 61% of reads were 48 bases and the mean length was 44.5 bases. The percentage of 35 bp reverse reads that mapped was very low with both alignment programmes (<2%) and these data were not used. The percentage of exon sequence covered by at least one read was slightly higher (64%) than that of the genome as a whole, which is consistent with exons occurring in more highly conserved regions of the genome.

### SNP discovery and annotation

**Figure 1:**
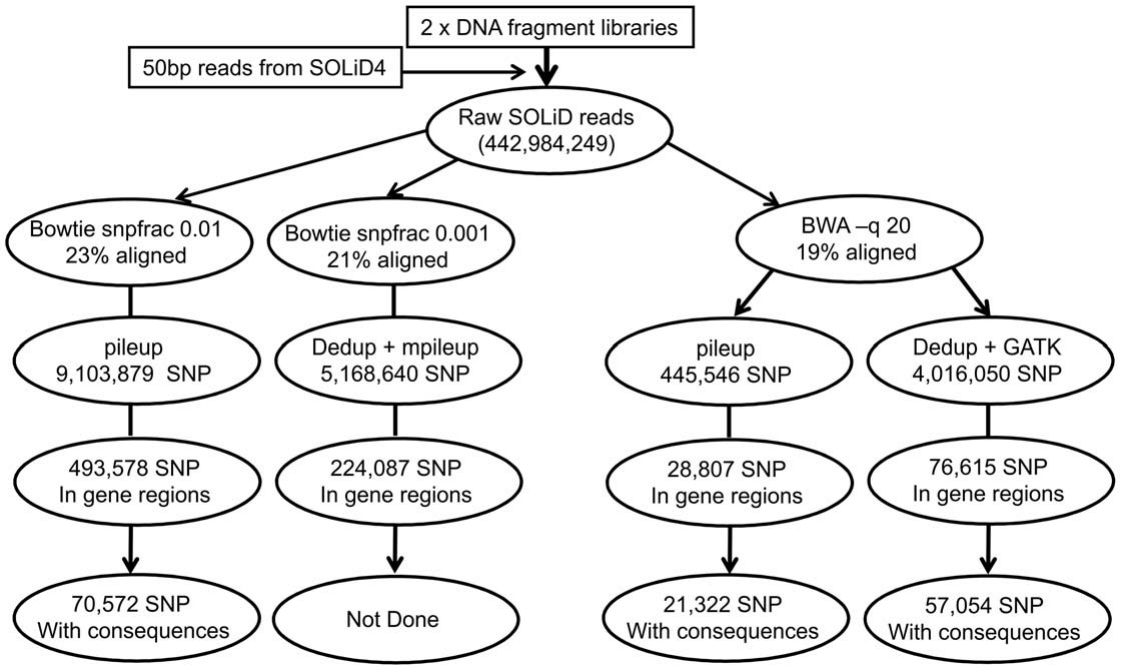
SNP discovery pipeline using Bowtie and BWA.

In order to be identified as SNPs, nucleotide variants called by SAMtools had to be supported by at least two reads, and have a base quality of at least 20, indicating 99% accuracy in the call. SNPs identified by GATK had to be classified as ‘PASS’. A flow diagram of the analysis is shown in Figure 1. The number of SNPs called by different pipelines varied substantially, with BWA followed by pileup having about a tenth of the other methods (Figure 1 and Table 1). GATK was run on the same BWA generated bam files as pileup and found 7 times more SNPs, so the difference in this case was due to the SNP calling and not the mapping. Bowtie and mpileup generated about half the number of SNPs as Bowtie and pileup; however, in this case Bowtie was run with snpfrac at 0.001 and 0.01 respectively and Picard tools was used to remove duplicates prior to mpileup but not pileup, so it is likely that the number of SNPs observed was reduced at several points in the pipeline leading to mpileup compared to that leading to pileup.

**Table 1:** Comparison of SNPs identified after mapping with BWA and Bowtie.

Number of SNPs	BWA pileup	BWA GATK	Bowtie pileup	Bowtie mpileup
Relative to reference sequence	445,546	3,739,203	6,881,214	4,234,692
Within buffalo population	109,361	276,847	2,222,665	933,948
Within gene regions	28,807	76,615	493,578	224,087

Gene regions are from the gene start to the gene end and include introns.

### SNP validation

173 SNPs were sent for validation on 87 Cape buffalo DNA samples, and 146 were amplified successfully. Of these 146 SNPs, 71 appeared to be monomorphic and 75 were polymorphic. The different mapping and SNP calling methods produced different numbers of validated SNPs (Table 2). The difference in validation rates between the three software packages was statistically significant (Chi sq test, χ 28, 4df, p<0.001). BWA followed by SAMtools Pileup or GATK did not detect 20 or 7 SNPs respectively that were detected by Bowtie and Pileup and validated as polymorphic. SNPs called by GATK were scored as “PASS” or “LowQual”; there was no difference in validation rate between these two classes of SNPs (ChiSq = 0.6, p = 0.45). Validated SNPs and KBioscience assay IDs can be seen in Table S1. Given the higher validation rate from the BWA data, this data was used for the subsequent analysis. An unbiased pattern of substitution would be expected to show a transition/transversion (Ti/Tv) ratio of 0.5, as transversions are twice as likely as transitions. However, a ratio of around 2.1 is usually observed in mammals, and a ratio that is significantly lower than 2.1 can be an indicator of poor quality sequencing data [23]. The observed Ti/Tv ratio for homozygous SNPs in this study was 2.18. Therefore, despite the relatively low validation rate, the Ti/Tv ratio suggested that SNP calls were in the expected proportions and were therefore not random.

**Table 2:** Counts of SNP that converted to successful assays predicted by different pipelines.

Validation result	BWA pileup	BWA GATK	Bowtie pileup	Bowtie mpileup
Monomorphic	20 (12)	31 (23)	71 (11)	46 (21)
Polymorphic	45 (6)	68 (12)	75 (4)	73 (13)
Not amplified. Number, (%)	12, 16% (1)	20, 17% (1)	27, 16% (0)	23, 13% (0)
Validated %	58%	57%	43%	51%
Percent of assays correctly identified as polymorphic	58% (67%)	57% (67%)	43% (45%)	54% (74%)

Figures in brackets are the number of loci that were predicted monomorphic (fixed for alternate allele) by the corresponding pipeline. The percentage correctly identified as polymorphic shows the percentage of all assays that were polymorphic and in brackets the percentage of assays predicted polymorphic that were confirmed.

### SNP Consequences

The predicted consequences of the SNPs were obtained from Ensembl and a summary of the numbers of the different types of consequences obtained using the BWA and GATK pipeline is shown in Table 3. 11,054 SNP had multiple annotations either because they were in multiple transcripts or because a SNP had more than one consequence e.g. SPLICE_SITE and INTRONIC and 3,105 SNP within gene regions had no annotation. A complete list of SNPs in exons with consequences is shown in Table S2. DAVID was used to discover whether any specific pathways were particularly affected by premature stop codons but none were significantly overrepresented after correction for multiple testing (minimum p value = 0.706) suggesting that these genes were affected at random. A web page was created where SNPs could be filtered by genomic region, gene, SNP consequence or restriction site (http://www.genomics.liv.ac.uk/tryps/resources.html) [28]. The inclusion of the restriction sites affected makes it possible to obtain lists of SNPs that can be used in RFLP assays. These are very useful where a set of markers is required across a genome region but the particular SNPs assayed are not important. The website implements Primer3 [29] to design primers around SNPs according to user-specified criteria.

**Table 3:** Counts of BWA/GATK SNPs of each consequence within gene regions.

SNP CONSEQUENCE	COUNT
DOWNSTREAM	3,400
STOP_LOST	1
UPSTREAM	3,265
INTRONIC	67,542
WITHIN_MATURE_miRNA	1
STOP_GAINED	26
SYNONYMOUS_CODING	1,408
SPLICE_SITE	205
NON_SYNONYMOUS_CODING	982
3PRIME_UTR	690
5PRIME_UTR	54
WITHIN_NON_CODING_GENE	106
ESSENTIAL_SPLICE_SITE	8

### Signatures of selection

Genes were screened for particularly high or low non-synonymous/synonymous substitution ratios (dN/dS) that might be indicative of positive or negative selection, respectively (Table S3). No genes had dN/dS ratios that were significantly higher than 1 (χ^2^ p<0.05). DAVID analysis of the 205 genes with no synonymous SNPs suggested that C-type lectins might be overrepresented in this set (Table S4). In contrast 1,231 genes had dN/dS ratios significantly less than 1 (p<0.05) suggesting that they were more conserved than expected. Genes involved in the regulatory processes “nucleoside binding” and “phosphorylation” were the most overrepresented (Table S5).

## Discussion

The African buffalo has become a species of interest in recent years due to its role as a wildlife maintenance host for a variety of infectious and zoonotic diseases, such as corridor disease, foot-and-mouth disease and bovine tuberculosis [13]. There is no African buffalo reference genome available for use in disease association studies, and much benefit would be gained by the generation of sequence data and large-scale SNP discovery in this species. The cost efficiency, large data output and fast turnaround time of the next-generation sequencing technologies have greatly facilitated the generation of novel sequence data as well as large-scale SNP discovery in non-model organisms. ABI SOLiD technology was used in this study to generate over 400 million 50 bp reads of African buffalo genome sequence, and for the preliminary identification of approximately a quarter of a million novel SNPs within the buffalo genome. When investigating a species for which a complete genome sequence is not available, the reference genome of a related species can be used for mapping and SNP discovery. The success of this approach was recently shown by mapping sequence reads of the great tit, *Parus major*, to the reference genome of the zebra finch, *Taeniopygia guttata* [10]. Using this method, the authors were able to identify 20,000 novel SNPs. A similar approach was used in mapping sequence reads of the turkey, *Meleagris gallopavo*, to the sequenced genome of the chicken, and approximately 8000 SNPs were identified in the turkey genome [30].

In this study, the Cape buffalo sequence reads were mapped to the reference genome of the domestic cow, *Bos taurus*. The most recent common ancestor of the domestic cow and the African buffalo is estimated to have existed approximately 5–10 million years ago (MYA), at the time of the divergence of the subtribes Bubalina, which consists of the *Syncerus* and *Bubalus* genera, and Bovina, which is comprised of the *Bos* and *Bison* genera [31]. Despite the relatively recent split of these two genera, only 19% to 23% of the buffalo short reads mapped to the reference cow genome using BWA and Bowtie. These percentages are comparable to those achieved by van Bers *et al*. [10], where 26% and 32% of the great tit sequence reads generated in two pools were mapped to the zebra finch genome using data generated by the Illumina Genome Analyser. Similarly, Kerstens *et al*. [30] mapped approximately 30% of the raw sequence reads generated in the turkey to the chicken genome. The low percentage mapping resulted in a depth of coverage that was substantially below a level that would be ideal for SNP discovery. It would appear that 2–4 times more raw data is required when using a distant relative as a reference sequence, although the longer reads that are now available from the Illumina sequencers (100–150 bp) might improve the percentage mapping. Nevertheless the extra data required when using a distant relative is still substantially less than would be required for a *de novo* assembly (>80× coverage), and mapping to a related species dramatically simplifies the SNP discovery and annotation compared to that required for a *de novo* assembly.

Significantly different results were found in this study when using different mapping and SNP calling methods (Table 1). From the combined BWA pileup and Bowtie pileup SNP pool, 173 SNPs from within the Cape buffalo population were selected for validation. The selection was based on gene function and SNP consequence, in order to use the validation data in a subsequent case-control association study. Validation required SNPs to pass two tests 1) that the loci amplified and 2) that the loci were polymorphic in Cape buffalo. The number of SNPs detected by each method that were validated ranged from 45–75, and the percentage ranged from 43–58% (Table 2). BWA and GATK had highest percent validated (57%), although it is probably not statistically significantly better than the Bowtie/mpileup combination (54% validated), which yielded more SNPs. The BWA/GATK SNPs were used for the functional analyses discussed below. It is not possible to accurately estimate false positive rates for any method except Bowtie and pileup since all SNPs assayed were originally identified with this method. The identification of false positives in SNP discovery may be a result of sequencing errors, alignment errors or the occurrence of paralogous sequence variants [1]. The error rate of the ABI SOLiD is estimated at 0.0006% [32], which would lead to about 50,000 erroneous base calls or 10% of the BWA and 0.5% of the Bowtie SNPs; in either case this is insufficient to explain the observed error rate, particularly since the Ti/Tv ratio was 2.18 as would be expected for a mammalian genome, rather than a rate nearer 0.5 which would be expected from random SNP calls. Therefore it seems more likely that the errors resulted from the low coverage (2.7×) and problems with aligning to the genome of a different species [33]. Since selected SNPs from this study will be used in future candidate gene association studies, it was desirable to identify as many SNPs as possible in order to obtain the largest number of candidates, and therefore false positives were considered less problematic than false negatives. SNPs within genes were annotated using the Ensembl SNP annotation API. Although this provided consequences for 57,054 SNPs, no general conclusions could be drawn from the data. There were no pathways overrepresented amongst the genes associated with the 27 SNPs that modified stop codons. It should be noted that the 1000 genomes project found 250–300 loss of function variants within genes in each individual [34], so the discovery of 27 in a pool of DNA from nine individuals probably does not have significant biological implications.

The non-synonymous/synonymous substitution ratios provided very little evidence for genes being under positive selection, although a large number appeared to be under purifying selection. This may be because this approach was developed for comparing distantly related taxa, and the relatively small number of SNPs between the Cape buffalo and the cow (mean 4.2 per gene) means that the power to detect positive selection is very limited [35]. The large number of genes that are apparently under purifying selection in the buffalo may contain many that are too slowly evolving to exhibit a detectable signal between these two species. Housekeeping genes appeared to be overrepresented in this list but this may be a consequence of their tendency to be relatively slowly evolving rather than evidence of purifying selection.

This investigation of the Cape buffalo, a species without an assembled genome, has yielded a wealth of data that will provide useful tools for further study of this species and related aspects.

## Supporting Information

Table S1 All data based on BWA mapping.
List of the 69 validated SNPs, their Btau4 and UMD3 positions and KBioscience assay IDs, ValidatedSNPs.txt.
(TXT)

Table S2 Complete list of BWA/GATK SNPs with consequences and annotated gene names, BwaSNPwithConsequences.txt.
(ZIP)

Table S3 List of genes that had at least one synonymous or non-synonymous SNP, with dN/dS ratios and p values calculated with PAML, Annotated_dNdSratios.txt.
(TXT)

Table S4 David annotation of genes with dN/dS ratios of 99, which are mainly those with no synonymous SNP, DAVIDAnalysisDnDS = 99.
(TXT)

Table S5 David annotation of genes that had dN/dS ratios significantly less than 1, DAVIDAnalysisDnDS_P0.05.
(TXT)

## References

[1] Garvin MR, Saitoh K, Gharrett AJ (2010) Application of single nucleotide polymorphisms to non-model species: a technical review. Molecular Ecology Resources 10: 915–934 doi:10.1111/j.1755-0998.2010.02891.x. 21565101 10.1111/j.1755-0998.2010.02891.x

[2] Shendure J, Ji H (2008) Next-generation DNA sequencing. NatBiotechnol 26: 1135–1145. 10.1038/nbt1486 18846087

[3] Seeb JE, Carvalho G, Hauser L, Naish K, Roberts S, et al (2011) Single-nucleotide polymorphism (SNP) discovery and applications of SNP genotyping in nonmodel organisms. Molecular Ecology Resources 11: 1–8 doi:10.1111/j.1755-0998.2010.02979.x. 21429158 10.1111/j.1755-0998.2010.02979.x

[4] Imelfort M, Duran C, Batley J, Edwards D (2009) Discovering genetic polymorphisms in next-generation sequencing data. Plant BiotechnolJ 7: 312–317. 19386039 10.1111/j.1467-7652.2009.00406.x

[5] Tautz D, Ellegren H, Weigel D (2010) Next Generation Molecular Ecology. Molecular Ecology 19: 1–3 doi:10.1111/j.1365-294X.2009.04489.x. 10.1111/j.1365-294X.2009.04489.x 20331765

[6] Voelkerding KV, Dames SA, Durtschi JD (2009) Next-Generation Sequencing: From Basic Research to Diagnostics. Clin Chem 55: 641–658 doi:10.1373/clinchem.2008.112789/. 19246620 10.1373/clinchem.2008.112789

[7] Metzker ML (2010) Sequencing technologies - the next generation. NatRevGenet 11: 31–46. 10.1038/nrg2626 19997069

[8] Morin PA, Luikart G, Wayne RK (2004) the SNP Workshop Group (2004) SNPs in ecology, evolution and conservation. Trends in Ecology & Evolution 19: 208–216 doi:16/j.tree.2004.01.009.

[9] Collins LJ, Biggs PJ, Voelckel C, Joly S (2008) AN APPROACH TO TRANSCRIPTOME ANALYSIS OF NON-MODEL ORGANISMS USING SHORT-READ SEQUENCES. Genome Informatics 2008 - Proceedings of the 19th International Conference. Gold Coast, Queensland, Australia. pp. 3–14. Available: http://eproceedings.worldscinet.com/9781848163324/9781848163324_0001.html. Accessed 19 July 2011. 19425143

[10] van Bers NEM, Oers KV, Kerstens HHD, Dibbits BW, Crooijmans RPMA, et al (2010) Genome-wide SNP detection in the great tit Parus major using high throughput sequencing. Molecular Ecology 19: 89–99 doi:10.1111/j.1365-294X.2009.04486.x. 20331773 10.1111/j.1365-294X.2009.04486.x

[11] Everett MV, Grau ED, Seeb JE (2011) Short reads and nonmodel species: exploring the complexities of next-generation sequence assembly and SNP discovery in the absence of a reference genome. Molecular Ecology Resources 11: 93–108 doi:10.1111/j.1755-0998.2010.02969.x. 21429166 10.1111/j.1755-0998.2010.02969.x

[12] Jolles AE (2007) Population biology of african buffalo (Syncerus caffer) at Hluhluwe-iMfolozi Park, South Africa. African Journal of Ecology 45: 398–406.

[13] Grobler JP, Van der Bank FH (1996) Genetic diversity and isolation in African buffalo (Syncerus caffer). Biochemical Systematics and Ecology 24: 757–761.

[14] Syncerus caffer (African Buffalo) (n.d.). Available:http://www.iucnredlist.org/apps/redlist/details/21251/0. Accessed 13 July 2011.

[15] van Hooft WF, Groen AF, Prins HH (2002) Phylogeography of the African buffalo based on mitochondrial and Y-chromosomal loci: Pleistocene origin and population expansion of the Cape buffalo subspecies. MolEcol 11: 267–279. 10.1046/j.1365-294x.2002.01429.x 11856427

[16] van Hooft WF, Groen AF, Prins HHT (2003) Genetic structure of African buffalo herds based on variation at the mitochondrial D-loop and autosomal microsatellite loci: Evidence for male-biased gene flow. Conservation Genetics 4: 467–477.

[17] Heller R, Lorenzen ED, Okello JB, Masembe C, Siegismund HR (2008) Mid-Holocene decline in African buffalos inferred from Bayesian coalescent-based analyses of microsatellites and mitochondrial DNA. MolEcol 17: 4845–4858. 10.1111/j.1365-294X.2008.03961.x 19140976

[18] Zimin AV, Delcher AL, Florea L, Kelley DR, Schatz MC, et al (2009) A whole-genome assembly of the domestic cow, Bos taurus. Genome Biol 10: R42 doi:10.1186/gb-2009-10-4-r42. 19393038 10.1186/gb-2009-10-4-r42 PMC2688933

[19] Li H, Durbin R (2009) Fast and accurate short read alignment with Burrows-Wheeler transform. Bioinformatics 25: 1754–1760 doi:10.1093/bioinformatics/btp324. 19451168 10.1093/bioinformatics/btp324 PMC2705234

[20] Langmead B, Trapnell C, Pop M, Salzberg SL (2009) Ultrafast and memory-efficient alignment of short DNA sequences to the human genome. Genome Biol 10: R25 doi:10.1186/gb-2009-10-3-r25. 19261174 10.1186/gb-2009-10-3-r25 PMC2690996

[21] McKenna A, Hanna M, Banks E, Sivachenko A, Cibulskis K, et al (2010) The Genome Analysis Toolkit: A MapReduce framework for analyzing next-generation DNA sequencing data. Genome Res 20: 1297–1303 doi:10.1101/gr.107524.110. 20644199 10.1101/gr.107524.110 PMC2928508

[22] Li H, Handsaker B, Wysoker A, Fennell T, Ruan J, et al (2009) The Sequence Alignment/Map format and SAMtools. Bioinformatics 25: 2078–2079 doi:10.1093/bioinformatics/btp352. 19505943 10.1093/bioinformatics/btp352 PMC2723002

[23] DePristo MA, Banks E, Poplin R, Garimella KV, Maguire JR, et al (2011) A framework for variation discovery and genotyping using next-generation DNA sequencing data. Nature Genetics 43: 491–498 doi:10.1038/ng.806. 21478889 10.1038/ng.806 PMC3083463

[24] Rios D, McLaren WM, Chen Y, Birney E, Stabenau A, et al (2010) A database and API for variation, dense genotyping and resequencing data. BMC Bioinformatics 11: 238 doi:10.1186/1471-2105-11-238. 20459810 10.1186/1471-2105-11-238 PMC2882931

[25] McLaren W, Pritchard B, Rios D, Chen Y, Flicek P, et al (2010) Deriving the consequences of genomic variants with the Ensembl API and SNP Effect Predictor. Bioinformatics 26: 2069–2070 doi:10.1093/bioinformatics/btq330. 20562413 10.1093/bioinformatics/btq330 PMC2916720

[26] Yang Z (1997) PAML: A Program Package for Phylogenetic Analysis by Maximum Likelihood. Comput Appl Biosci 13: 555–556 doi:10.1093/bioinformatics/13.5.555. 9367129 10.1093/bioinformatics/13.5.555

[27] Dennis G, Sherman BT, Hosack DA, Yang J, Gao W, et al (2003) DAVID: Database for Annotation, Visualization, and Integrated Discovery. Genome Biol 4: R60–R60. PMC193660 12734009

[28] Noyes H (2011) Cape Buffalo SNP. Available: http://www.genomics.liv.ac.uk/tryps/capeSNP.php.

[29] Rozen S, Skaletsky H (2000) Primer3 on the WWW for general users and for biologist programmers. Methods Mol Biol 132: 365–386. 10547847 10.1385/1-59259-192-2:365

[30] Kerstens H, Crooijmans R, Veenendaal A, Dibbits B, Chin-A-Woeng T, et al (2009) Large scale single nucleotide polymorphism discovery in unsequenced genomes using second generation high throughput sequencing technology: applied to turkey. BMC Genomics 10: 479 doi:10.1186/1471-2164-10-479. 19835600 10.1186/1471-2164-10-479 PMC2772860

[31] MacEachern S, McEwan J, Goddard M (2009) Phylogenetic reconstruction and the identification of ancient polymorphism in the Bovini tribe (Bovidae, Bovinae). BMC Genomics 10: 177 doi:10.1186/1471-2164-10-177. 19393045 10.1186/1471-2164-10-177 PMC2694835

[32] Applied Biosystems (2010) Applied Biosystems SOLiD4 System. Available: http://www3.appliedbiosystems.com/cms/groups/mcb_support/documents/generaldocuments/cms_080173.pdf.

[33] Morozova O, Marra MA (2008) Applications of next-generation sequencing technologies in functional genomics. Genomics 92: 255–264 doi:16/j.ygeno.2008.07.001. 18703132 10.1016/j.ygeno.2008.07.001

[34] Genomes Project Consortium (2010) A map of human genome variation from population-scale sequencing. Nature 467: 1061–1073 doi:10.1038/nature09534. 20981092 10.1038/nature09534 PMC3042601

[35] Kryazhimskiy S, Plotkin JB (2008) The Population Genetics of dN/dS. PLoS Genet 4: e1000304 doi:10.1371/journal.pgen.1000304. 19081788 10.1371/journal.pgen.1000304 PMC2596312

